# Epicardial adipose tissue thickness is related to early subclinical myocardial dysfunction, particularly in patients with type 2 diabetes mellitus: a case control study

**DOI:** 10.1186/s12872-022-02944-8

**Published:** 2022-12-02

**Authors:** David Eduardo Prestegui-Muñóz, Daniel Rabindranath Benítez-Maldonado, Karen Rodríguez-Álvarez, José Ángel de Jesús Prestegui-Muñoz, Alberto Melchor-López, Juan Antonio Suárez-Cuenca

**Affiliations:** 1 Hospital General de Ticomán, Secretaría de Salud Ciudad de México, Mexico City, Mexico; 2 Escuela Nacional de Medicina y Homeopatía, Instituto Politécnico Nacional, Mexico City, Mexico; 3 Hospital General Xoco, Secretaría de Salud Ciudad de México, Mexico City, Mexico

**Keywords:** Epicardial adipose thickness, Strain, Diabetes mellitus

## Abstract

**Background:**

Cardiac myofibrillary dysfunction, which can be measure by echocardiographical s*train value,* represents an early subclinical manifestation of heart failure. Epicardial Adipose tissue (EAT) is related to low degree inflammation and oxidative damage in the adjacent tissue.

**Aim:**

To explore whether EAT affects early myocardial dysfunction, as assessed *strain* values.

**Methods:**

Case–Control design. Patients lacking clinical significant heart failure, thyroid or renal disease or malignant abnormalities were included. Clinical-demographic and biochemical data were collected. EAT and myofibril deformation were measured by echocardiography.

**Results:**

A total of 71 patients were analyzed, and further subdivided according to type 2 Diabetes Mellitus (t2DM). Higher *strain* value (higher than -22.4%cut-off value) was associated with male sex and higher anthropometric and metabolic risk measures; particularly those with t2DM. Higher EAT was also associated higher *strain* value (AUC = 0.92 ± 0.06, *p* = 0.004), and further correlation was evidenced (rho = 0.488, *p* < 0.001), with significant influence of t2DM.

**Conclusion:**

EAT was related to *strain* value, suggesting the influence of cardiac adipose tissue on the deformability of cardiac myofibril, with a more significant effect in the population with t2DM.

## Background

Cardiac myofibrillary dysfunction is an early subclinical manifestation of heart failure, and precedes a significant reduction of the left ventricle ejection fraction. Such myofibrillary dysfunction may be measure through an echocardiographical parameter known as the “*strain*”, which can be described as the normalized change in length between two points of the myofibril, evaluated in a segment of the myocardium [1]; whereas the grade of deformation is generally expressed as percentage. The more negative *strain* value the better myofibril shortening. In the clinical practice, longitudinal *strain* (deformation in the base-apex direction) is useful for evaluating left ventricle contractile function [2]. Some cardiometabolic conditions like type 2 Diabetes Mellitus (t2DM), insulin resistance or obesity may induce cardiac myofibrillary dysfunction [3–5].

Epicardial adipose tissue (EAT) is a type of visceral fat that shares similar embryonic origin than intra-abdominal visceral adipose tissue. EAT lays at the external wall of the myocardium and maintains intimal contact with the epicardial vessels, sharing the same microcirculation coming from branches of the coronary arteries [6].

EAT shows compressibility and elasticity that are important to provide mechanical protection to the coronary arteries against the excessive distortion caused by the arterial pulse and myocardial contraction [7]. EAT has been considered a metabolically active tissue and has bseen proposed as an emerging cardiovascular risk factor. Under normal conditions, EAT is a source of anti-atherogenic and anti-inflammatory adipocytokines, which prevent the myocardium from being exposed to high levels free fatty acids (FFA) [8]. Nevertheless, the increase of EAT thickness, as measured by echocardiography has been proposed as a cardiometabolic risk factor, independent from either the abdominal visceral fat or BMI [9]. Narváez et al. showed a significant relationship between EAT > 3 mm with the presence of metabolic syndrome [10, 11].

Currently, it is not clear whether EAT may relate with cardiac myofibrillar dysfunction. Therefore, the aim of this study was to explore whether EAT impacts early myocardial dysfunction and the influence of comorbidities.

## Methods

### Design and study population

#### Cross-sectional design

The present study was carried out from June 2019 to February 2020 at Ticoman General Hospital, Mexico City. Subjects participating were adults older than 18 years old, with t2DM, who were attended at Internal Medicine outpatient Clinic; as well as healthy subjects, age- sex- matched. Participants were excluded if they presented additional co-morbidity, malignant disease, drug use or pregnancy.

All cases were de-identified to comply with data protection recommendation for research. This project was registered and approved by the Institutional Boards of Ethics and Research, Ticoman General Hospital, SEDESA, Mexico City (approval ID 207.010.30.18) and all the experiments were performed according to Health General Law—National Guideline, in accordance to the Good Clinical Practices Guidelines as well as the Ethics recommendation from Helsinki declaration. All participants, and/or their legal guardian(s), signed informed consent before have been recruited.

#### Clinical and biochemical data collection

Clinical-demographic data were collected during initial interview. Anthropometric data like weight, height and waist circumference were registered, and body mass index (BMI) was calculated. A blood sample was obtained by vein puncture after a 12-h fast, and plasma lipids, glycemia and HbA1c were determined by routine automatized laboratory analyzer.

#### EAT and strain measures

Echocardiographic, long parasternal axis was used to measure EAT as follows. EAT was measure at free wall of the right ventricle. Determination was performed at the end of the systole, and 3 different determinations were recorded at 3 consecutive cardiac cycles, and calculating the EAT average, as described by Dr Iacobellis et al. [12]. The measurements of *strain* and *strain rate* were performed using tissue Ultrasound Doppler mode at 4 chamber, apical axis, using a 3.5 MHz transducer, Aloka alfa 6 equipment (Japan). Image captures were obtained from 3 cardiac cycles. Either EAT or Doppler determinations were simultaneously performed by two experienced cardiologist, unaware of the clinical data of the patients.

### Statistical analysis

Kolmogórov-Smirnov test was used to determine normality of data distribution. Quantitative data were presented as mean and standard deviation or median and interquartile range appropriately. Qualitative data were expressed as n (%). Statistical comparisons included student T-test or Mann–Whitney test, as well as Pearson correlation. ROC analysis was used to estimate EAT cutoff value, then Odds Ratio (OR) risk and multiple regression analysis were performed. Statistical analysis was performed using the SPSS v.25.0. A *p*-value ≤ 0.05 was considered as statistical significant.

## Results

The study population was constituted by 71 patients, mean aged 49.0 ± 9.3 years old, paired by gender (36 males, 35 females); mean HbA1c 7.4%, c-LDL 110.7 and mean EAT of 4.2 ± 1.2 mm.

For comparison purpose, the study population was divided according to the *Strain value* (-22.4% median cut-off value) and sub-analyses were performed by gender and T2DM (only females). In general, a higher *Strain* value was related with higher values of weight, BMI and SBP; as well as higher EAT (Table 1). Likewise, higher *strain* value was particularly distributed between females/T2DM and males/nonT2DM.

**Table 1:** Clinical-demographic characteristics of the study population (*n* = 71)

	***MALES (n* = *36)***			***FEMALES (n* = *35)***		
	***ALL MALES***	***DIVIDED BY STRAIN***		***ALL FEMALES***	***DIVIDED BY STRAIN***	
		***Strain* ≤ *-22.4% (Higher myofibrill deformation)*** (*n* = 14)	***Strain* > *-22.4% (Lower myofibrill deformation)*** (*n* = 22)		***Strain* ≤ *-22.4% (Higher myofibrill deformation)*** (*n* = 18)	***Strain* > *-22.4% (Lower myofibrill deformation)*** (*n* = 17)
**Age (years)**						
***All***	51 ± 9	48.7 ± 8.7	52.6 ± 9	46.9 ± 9	44.8 ± 10.4	49.18 ± 6.9
***non T2DM***	50 ± 9	48.7 ± 8.7	55.5 ± 9	44.5 ± 10	43.7 ± 10	48.3 ± 10.1
***t2DM***	52 ± 9	-	52.0 ± 9	49.5 ± 7	50.6 ± 10	49.3 ± 6.5
**Weight (Kg)**						
***All***	77 ± 14	75.3 ± 11	78.1 ± 16	67.3 ± 12	64.1 ± 12	70.8 ± 12
***non T2DM***	78 ± 13	75.3 ± 11	88.3 ± 16	67.9 ± 14	65.9 ± 13	77.6 ± 19
***T2DM***	75 ± 16	-	75.8 ± 16	66.8 ± 11	54.8 ± 3	69.3 ± 10**
**BMI (Kg/m**^**2**^**)**						
***All***	28.0 ± 4	27.0 ± 3	28.8 ± 5	26.8 ± 4	25.7 ± 4	28.0 ± 4
***non T2DM***	28.1 ± 4	27.0 ± 3	31.6 ± 4**	26.7 ± 4	23.3 ± 4	28.8 ± 6
***T2DM***	28.2 ± 5	-	28.2 ± 5	26.9 ± 4	22.8 ± 1	27.8 ± 3**
**Waist Circumference (cm)**						
***All***	105.0 ± 19	98.7 ± 10	109.2 ± 22	97.4 ± 17	92.8 ± 17	102.3 ± 18
***non T2DM***	101.0 ± 12	98.7 ± 10	111.0 ± 13	95.5 ± 18	94.4 ± 18	100.6 ± 18
***T2DM***	108.8 ± 24	-	108.0 ± 24	99.5 ± 17	84.6 ± 3	102.6 ± 18
**SBP (mmHg)**						
***All***	121.0 ± 12	113.6 ± 9	126.9 ± 12	119.2 ± 15	118.9 ± 15	119.5 ± 16
***non T2DM***	117.0 ± 11	113.6 ± 9	129.0 ± 8**	117.2 ± 13	117.4 ± 14	116.6 ± 15
***T2DM***	126.3 ± 13*	-	126.0 ± 13	121.3 ± 17	126.6 ± 25	120.2 ± 17
**DBP (mmHg)**						
***All***	75.0 ± 9	72.9 ± 11	77.9 ± 8	76.1 ± 8	76.0 ± 7	76.2 ± 9
***non T2DM***	74.0 ± 11	72.9 ± 11	78.5 ± 8	76.0 ± 6	75.5 ± 6	78.3 ± 8
***T2DM***	77.7 ± 8	-	77.7 ± 8	76.2 ± 9	78.3 ± 10	75.7 ± 9
**Glucose (mg/dL) Glucose**						
***All***	115.0 ± 52	105.5 ± 27	139.5 ± 60	135.6 ± 79	118.0 ± 61	154.1 ± 94
***non T2DM***	102.7 ± 25	105.5 ± 27	93.2 ± 5	116.1 ± 61	114.6 ± 65	123.3 ± 47
***T2DM***	149.7 ± 62*	-	149.0 ± 62	156.2 ± 93	135.0 ± 38	160.7 ± 101
**HbA1c (%)**						
***All***	7.1 ± 2	5.9 ± 0.37	7.8 ± 2.9	7.7 ± 2.4	6.6 ± 2.0	8.9 ± 3
***non T2DM***	5.9 ± 0	5.9 ± 0.37	6.1 ± 0.25	6.0 ± 0.5	6.0 ± 0.5	5.9 ± 1
***T2DM***	8.2 ± 3*	-	8.2 ± 3.1	9.5 ± 2.3*	9.5 ± 2.0*	9.5 ± 2*
**Cholesterol (mg/dL)**						
***All***	189.0 ± 47	179.6 ± 56	195.5 ± 42	195.0 ± 43	190.9 ± 46	199.7 ± 42
***non T2DM***	187.6 ± 52	179.6 ± 56	215.7 ± 26	195.7 ± 50	188.4 ± 49	232.6 ± 40
***T2DM***	191.0 ± 43	-	191.0 ± 43	194.6 ± 38	200.3 ± 22	192.7 ± 41
**Triglycerides (mg/dL)**						
***All***	177.0 ± 95	144.4 ± 113	198.8 ± 77	189.0 ± 120	160.8 ± 69	219.6 ± 155
***non T2DM***	154.0 ± 101	144.4 ± 113	187.0 ± 21	169.7 ± 70	160.2 ± 64	217.3 ± 92
***T2DM***	201.0 ± 85	-	201.0 ± 85	210.1 ± 157	163.5 ± 106	220.1 ± 168
**c-HDL (mg/dL)**						
***All***	45.7 ± 22	47.4 ± 28	44.6 ± 18	49.6 ± 20	52.4 ± 21	46.6 ± 20
***non T2DM***	47.5 ± 25	47.4 ± 28	47.9 ± 11	48.0 ± 17	49.7 ± 18	40.0 ± 11
***T2DM***	43.9 ± 20	-	43.9 ± 20	51.2 ± 24	66.1 ± 34	48.0 ± 21
**c-LDL (mg/dL)**						
***All***	109.3 ± 43	104.9 ± 46	112.1 ± 41	112.2 ± 38	115.5 ± 43	108.8 ± 34
***non T2DM***	107.6 ± 46	104.9 ± 46	116.0 ± 51	117.4 ± 42	114.9 ± 46	129.6 ± 9
***T2DM***	111.1 ± 41	-	111.0 ± 41	106.8 ± 35	118.5 ± 30	104.3 ± 36
**EAT**						
***All***	4.46 ± 1	3.4 ± 1	5.1 ± 1**	3.9 ± 1.1	3.62 ± 1.06	4.3 ± 1.2
***non T2DM***	3.43 ± 1	3.4 ± 1	3.5 ± 1	3.19 ± 0.83	3.28 ± 0.79	2.7 ± 1.1
***T2DM***	5.49 ± 1*	-	5.4 ± 1*	4.74 ± 0.88*	5.31 ± 0.43*	4.6 ± 0.91*
**STRAIN**						
***All***	-22.1 ± 2.4	-24.4 ± 2	-20.6 ± 0.93	-22.8 ± 2.9	-24.8 ± 2.7	-20.6 ± 0.64
***non T2DM***	-23.7 ± 2.2	-24.4 ± 2	-21.6 ± 0.69**	-24.6 ± 2.9	-25.2 ± 2.84	-21.4 ± 0.38**
***T2DM***	-20.3 ± 0.8*	-	-20.3 ± 0.84*	-20.9 ± 1.14*	-22.9 ± 0.19	-20.4 ± 0.68*

Quantitative data was analyzed by 2-tail, T-test, and Categorical data by X^2^

*Abbreviations*: *t2DM* type 2 Diabetes Mellitus*, SBP* Systemic blood pressure, *DBP* Diastolic blood pressure, *BMI* Body Mass Index, *HbA1c* Glycated hemoglobin, *cHDL* High-density Lipoprotein cholesterol, *cLDL* Low-density Lipoprotein cholesterol, *EAT* Epicardial Adipose Thickness

^*^p < 0.005 difference between non-T2DM vs DT2DM

^**^p < 0.005 difference between higher myofibrill deformation vs lower myofibrill deformation)

**Fig. 1:**
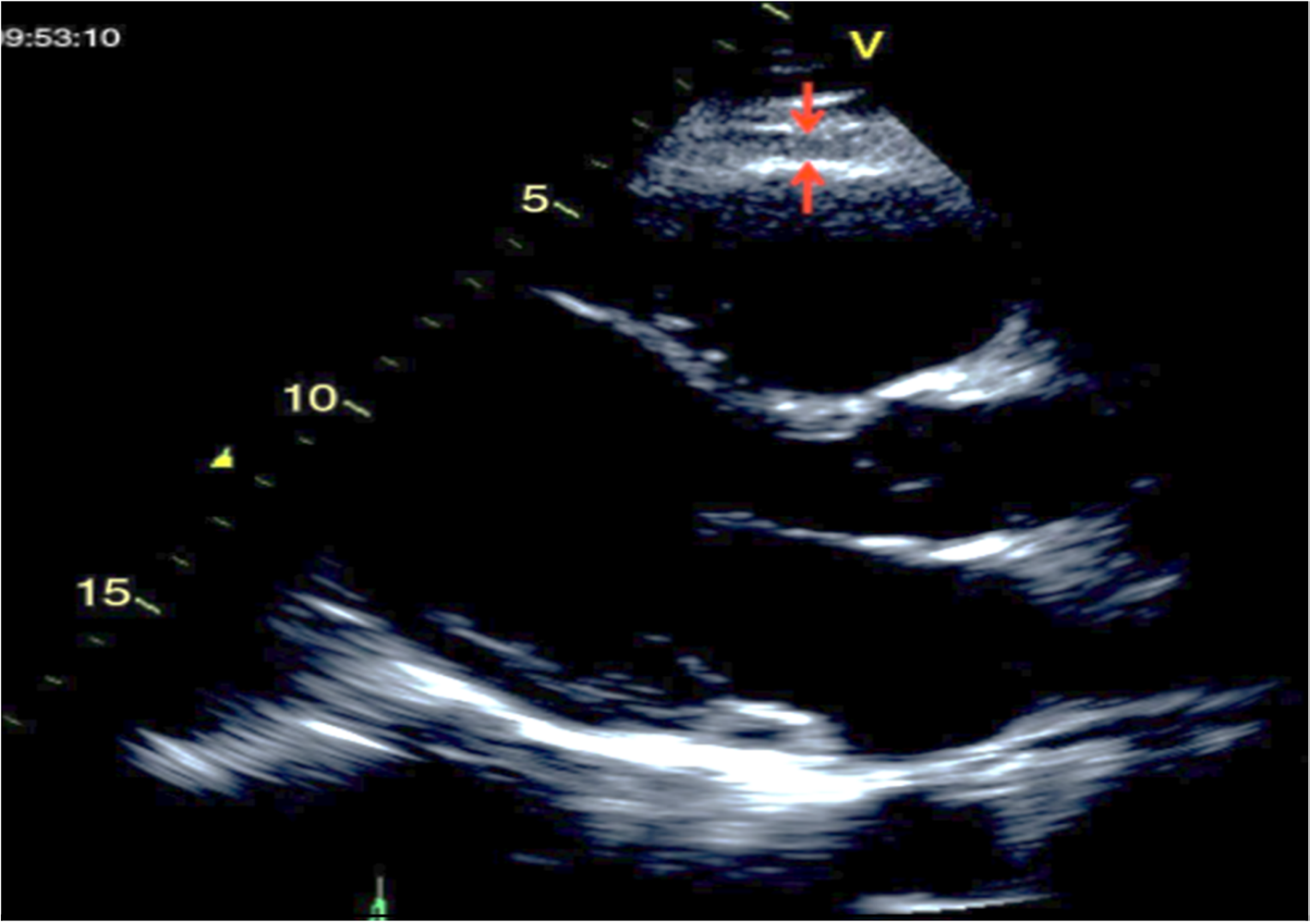
Epicardial Adipose Tissue Thickness. Echocardiographic measure of epicardial fat thickness (pointed by the red arrows) identified as the echofree space between the outer wall of the myocardium and the visceral layer of pericardium in the parasternal long-axis view

**Fig. 2:**
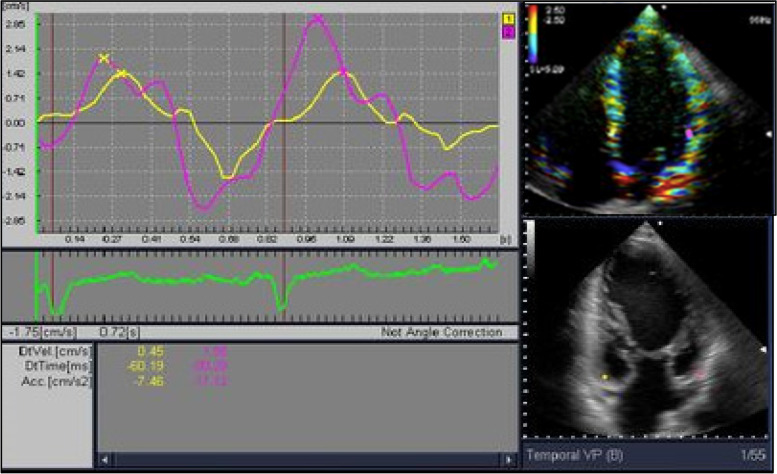
Strain measurement. Tissue Doppler echocardiography, strain rate, and strain profiles of a normal subject obtained from an apical four chamber view. Time is on the X-axis. The waveform consists of a first positive wave (pre-ejection period), followed by the isovolumic relaxation waves. Longitudinal strain rate profile is obtained from the same region of interest during the same cardiac cycle

**Fig. 3:**
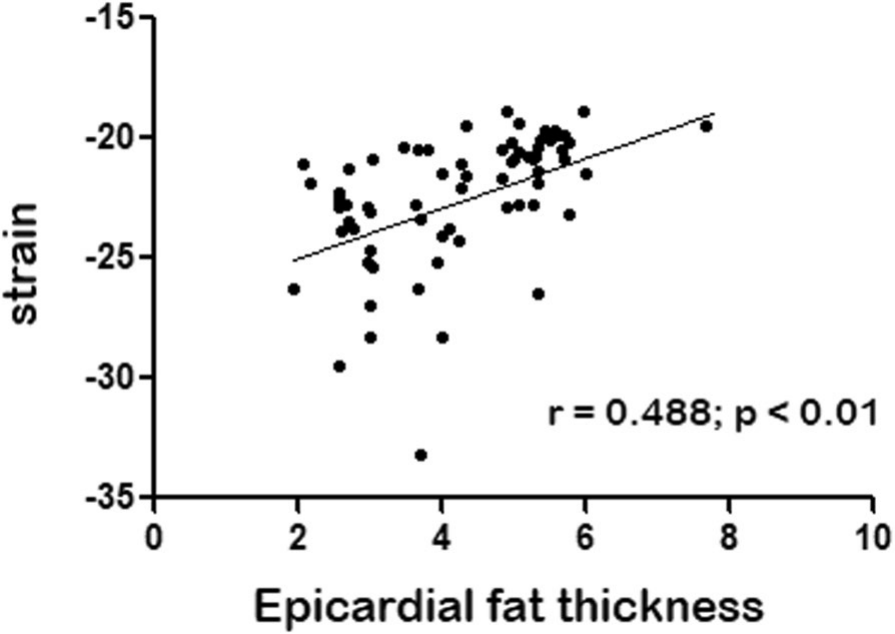
Strain and Epicardial Adipose Tissue. The figure shows the correlation between Strain and Epicardial Adipose Tissue Thickness

**Fig. 4:**
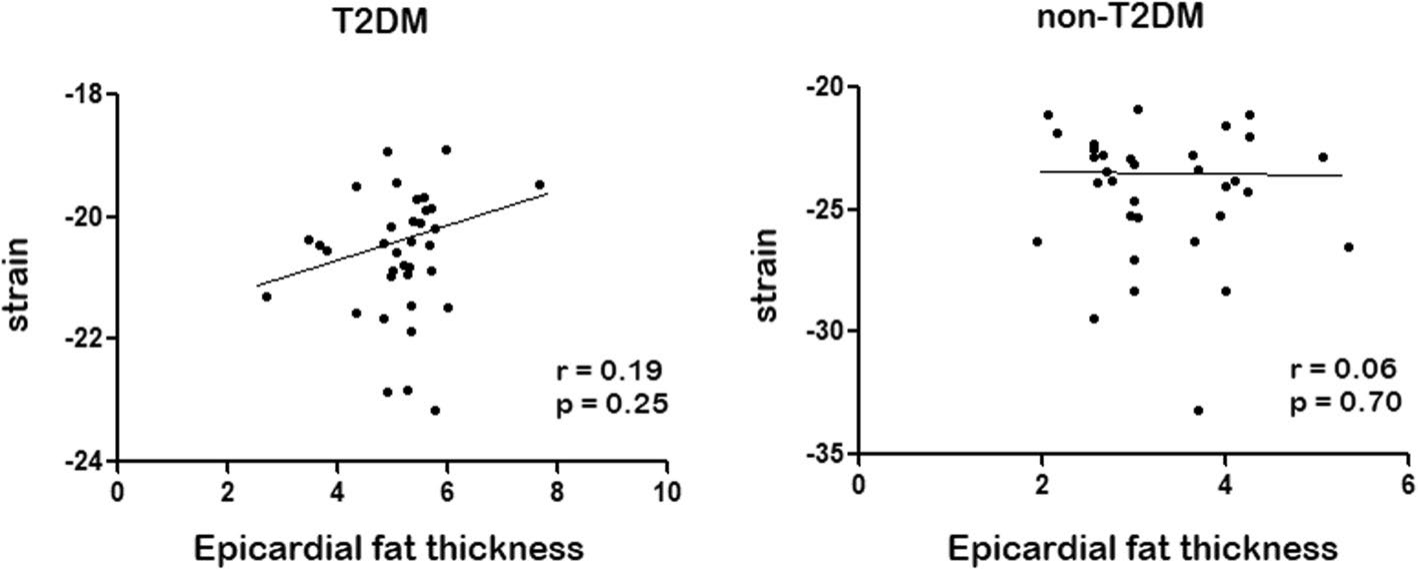
Strain, Epicardial Adipose Tissue and T2DM. The figure shows the correlation between Strain and Epicardial Adipose Tissue Thickness, further grouped in T2DM and non-T2DM. Abbreviatures: T2DM, type 2 Diabetes Mellitus

**Fig. 5:**
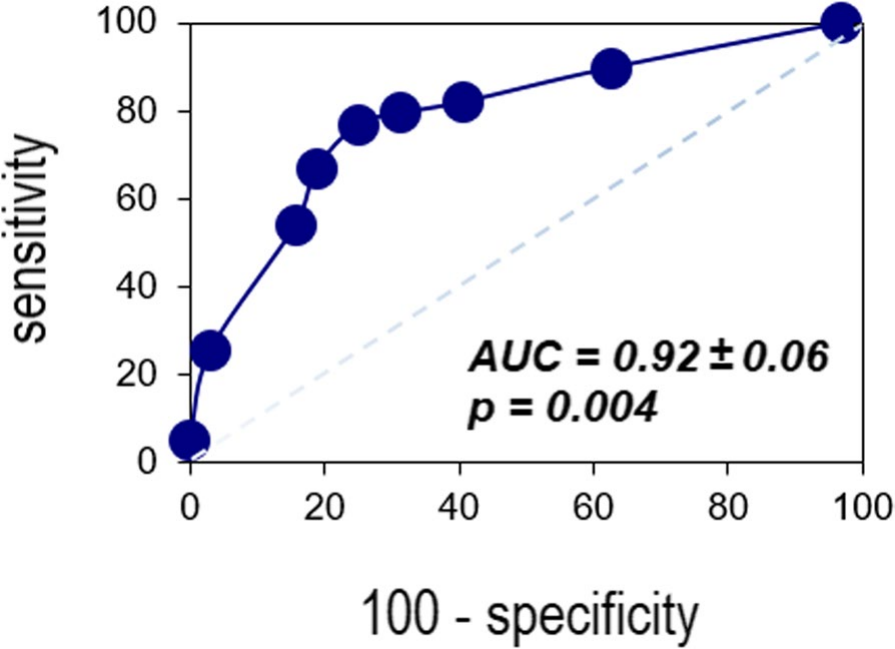
Diagnostic Performance of EAT for Strain Discrimination. ROC Curve analysis of EAT (cutoff value 4.1 mm) to discriminate Strain value lower than -22.4%. Abbreviations: AUC, Area Under the Curve

To further explore potential relation with biomarkers, EAT (Fig. 1) and *Strain* (Fig. 2) were correlated. A significant positive correlation was evidenced between EAT and *strain* value (*r* = 0.488, *p* < 0.001) (Fig. 3); while stratified correlation analysis showed significant differences between patients with or w/o t2DM (Fig. 4). Furthermore, EAT cutoff value of 4.1 mm was useful to discriminate *Strain* value lower than -22.4%, as estimated by ROC curve AUC = 0.92 ± 0.06, *p* = 0.004, sensitivity 76.9%, specificity 75% (Fig. 5).

Finally, logistic regression analysis showed that higher plasma LDL cholesterol tended to be associated with lower myocardial deformity (OR 2.3, 95%CI 0.8–6.1 *p* = 0.047), but not other well-known cardiovascular risk factors, such as gender (*p* = 0.20), BMI (*p* = 0.93), abdominal circumference (*p* = 0.85) or HbA1c (*p* = 0.89).

## Discussion

The main finding of the present study was the relationship between EAT and *Strain*, with significant differences between diabetic and non-diabetic patients.

This finding let us speculate that EAT may limit the deformation capacity of the cardiac myofibril. Consistently, Cho et al. found that EAT was associated with the degree of subclinical myocardial dysfunction (longitudinal *strain*), as well as with ventricular mass and high sensitivity C-reactive protein values [13]. Similarly, Arnold et al. reported a significant association between EAT and longitudinal *strain* in subjects with metabolic syndrome, obesity and coronary artery disease [14].

Epicardial fat could induce dysfunction of myofibril deformity by different processes: 1) mechanical impairment: the increase in the EAT would limit the movement of the myocardium, and 2) inflammation: epicardial fat has histological characteristics of brown fat, which is in close proximity with coronary arteries and has the potential ability to secrete pro-inflammatory adipokines and free fatty acids, which can produce atherosclerosis of the coronary microvasculature and ischemia of the myofibril [13, 14]. In addition, higher free fatty acids may facilitate intramyocardial triglycerides accumulation (steatosis) and induction of cellular oxidative stress, as well as the higher activity of nitric oxide synthase and intracellular production of nitric oxide, which leads to myofibril apoptosis.

To our knowledge, the present study is the first to evaluate the role of t2DM on the relation between EAT and longitudinal *strain*. In this regard, higher values of EAT in subjects with t2DM had previously been reported [10, 15, 16]; while we further observed that most of the subjects with EAT > 4.2 mm and concomitant t2DM showed a limited deformation capacity of the cardiac myofibril (Table 1).

Similar observation was performed by Zhang et al. during comparison between patients with t2DM, where non-controlled t2DM preceded higher *Strain* values in all spatial directions and controlled t2DM affected only longitudinal *Strain* [17].

Furthermore, deformity of myofibrill may be affected by several cardiometabolic risk factors, like weight, BMI and insulin resistance. Consistent with this statement, Liu et al., observed a reduction in left ventricle tension in hypercholesterolemic rabbits, in comparison with normal controls [18]. Likewise, Vitarelli et al., observed a higher deterioration of left ventricle’s myofibril deformation in obese/hypercholesterolemic children and adolescents [19].

Interestingly, multivariate analysis showed that EAT did not associate with *Strain* or HbA1c; but with t2DM. This is consistent with findings described by Xiaoling et al. where patients with t2DM had less myocardial deformity, being more evident in patients with HbA1c > 7% [14].

In the present study, the median value of the *Strain* was considered as the cut-off value for our analyses, which was lower than the *Strain* values reported in other studies: − 15.80% to − 23.40% [20]. Such difference could be explained by characteristics of the study population and/or the equipment and software used for the measurements. In addition, EAT cut-off value obtained through ROC analysis or median value, were both useful to discriminate lower myofibril deformity. This is useful since EAT measure may be less complex to obtain than *Strain* determination.

Limitations of our study include the low number of patients, the lack of T2DM subgroup in male analysis and technological restrictions related with the available echocardiographic equipment, which lacks specific software for *Strain* determination.

In conclusion, EAT significantly related to myofibril deformation, with additional influence of t2DM.

## Data Availability

The datasets generated and analyzed during the current study are not publicly available due to privacy policies of the hospital and patients information; but are available from the corresponding author on reasonable request.
